# The association between dietary patterns, plasma lipid profiles, and inflammatory potential in a vascular dementia cohort

**DOI:** 10.1002/agm2.12249

**Published:** 2023-04-01

**Authors:** Jun Dai, Daniel Kam Yin Chan, Richard O. Chan, Vasant Hirani, Ying Hua Xu, Nady Braidy

**Affiliations:** ^1^ Department of Aged Care and Rehabilitation Bankstown‐Lidcombe Hospital 2200 New South Wales Bankstown Australia; ^2^ Faculty of Medicine University of New South Wales 2052 New South Wales Sydney Australia; ^3^ School of Life and Environmental Sciences University of Sydney 2006 New South Wales Sydney Australia; ^4^ Present address: Department of Aged Care Central Gippsland Health Service 3850 Victoria Sale Australia; ^5^ Present address: Shop 18A, Oxford Village, 63 Oxford Street Sydney New South Wales 2010 Australia

**Keywords:** diet, inflammation, lifestyle factors, lipidomics, lipids, vascular dementia

## Abstract

**Background:**

Inflammation and altered lipid dyshomeostasis have been implicated in the pathogenesis of Alzheimer's disease and vascular dementia.

**Objective:**

To determine if there are any associations between dietary patterns, plasma lipid profiles, and inflammatory potential in a vascular dementia cohort.

**Methods:**

One hundred fifty participants (36 subjects with Vascular Dementia and 114 healthy controls) from two Australian teaching hospitals completed a cross‐sectional survey examining their dietary and lifestyle patterns. Each participant's diet was further evaluated using the Empirical Dietary Inflammatory Index. Some participants also donated blood samples for lipidomic analysis.

**Results:**

After adjusting for age, education, and socioeconomic status, participants with vascular dementia tend to have higher lipid profiles, do less exercise, and engage less frequently in social interaction, educational, or reading activities. They also tend to consume more deep‐fried food and full‐fat dairy compared to control subjects. However, there was no difference in Empirical Dietary Inflammatory Index between the two groups after adjusting for age, education, and socioeconomic status.

**Conclusion:**

Our findings suggest a graded inverse association between healthy lifestyle factors and vascular dementia.

## Graphical abstract

Thirty‐six subjects with vascular dementia and 114 controls completed a survey examining their diet and lifestyle. Adjusting for age, education, and socioeconomic status, participants with vascular dementia have higher lipid profiles, do less exercise, and engage less in social interaction, educational, or reading activities. They also consume more deep‐fried food and full‐fat dairy compared to controls.

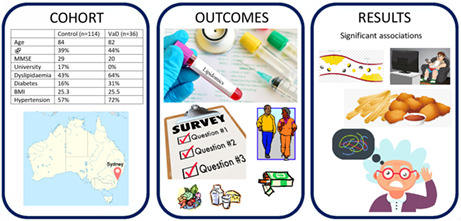

## 1 INTRODUCTION

Vascular dementia (VaD) is the second most common form of dementia after Alzheimer's disease (AD).^1^, ^2^ Inflammation and altered lipid dyshomeostasis have been implicated in the pathogenesis of AD and VaD.^3^

The Mediterranean and the Dietary Approaches to Stop Hypertension (DASH) diets have been associated with improved cognitive performance and reduced incident risk of dementia.^4^ These diets have been associated with reduced plasma lipid levels and lower plasma inflammatory markers.^5^ The Mediterranean diet involves regular intake of fruits and vegetables, whole grain products, low‐fat dairy products, olive oil, fish, poultry, and wine with meat. Similarly, the DASH diet comprises regular intake of fruits and vegetables, low‐fat dairy, legumes, nuts, and lower intake of animal proteins and sweets.

The aim of this study was to explore associations between lifestyle factors, diets, and their inflammatory potential, lipid profiles, and VaD. We hypothesize that individuals with VaD will have more pro‐inflammatory diets and lipid dysfunction compared with controls.

## 2 METHODS

### 2.1 Study participants

About 163 participants were screened for the study from the Bankstown‐Lidcombe Hospital and the War Memorial Hospital, Sydney, Australia. These participants were recruited from the two hospitals' community catchment area, memory disorder clinics, and inpatient wards from 1 January 2007 and 30 June 2020 as part of the Healthy Longevity project and Vascular Dementia project.^6^, ^7^, ^8^ After excluding participants with dementia of predominantly other causes (*n* = 11) at baseline and incomplete data for required variable (*n* = 2), a total of 114 controls and 36 VaD participants remained. Control participants (no VaD or dementia) were clinically determined to be cognitively normal with a Mini‐Mental State Examination (MMSE) score ≥ 28. Control participants with evidence of cerebrovascular events through history or on imaging were excluded. Regarding VaD, the inclusion criteria were: (1) age > 65 years; (2) meeting the diagnosis criteria of the National Institute of Neurological Disorders and Stroke (NINDS) and the Association Internationale pour la Recherce et l'Enseignement en Neurosciences (AIREN)^9^; (3) clinically diagnosed by an experienced geriatrician, a psychogeriatrician, and/or a neurologist. An independent geriatrician's opinion was sought for uncertain cases and such participants would only be included if two clinicians were in consensus of the VaD diagnosis; (4) at least one cerebral imaging modality—CT, SPECT, or MRI was needed to corroborate that the diagnosis was specifically small vessel VaD; and (5) the MMSE score was between 10 and 24 for the diagnosis of mild to moderate dementia. Exclusion criteria that applied to both control and VaD groups were: (1) current diagnosis of malignancy; and (2) presence of life‐threatening illnesses, acute psychiatric disorder; concomitant with AD component or other pathologies that were predominant etiology of dementia. We did not adjust MMSE scores for education.

Upon recruitment, participants completed a structured interview, including demographics, education level, diet, lifestyle factors, and medical conditions (hypertension, myocardial infarction, stroke, or diabetes mellitus) and additional information, including the longevity of a family member (father, mother, brother, or sister) and whether a family member had been diagnosed with cardiovascular, cerebrovascular or neurodegenerative disease, and dementia. Past medical history was collected from hospital discharge summaries and GP health summaries. Participants were examined and a blood sample was obtained. To analyze lipid profiles, fasting EDTA plasma was separated from whole blood. Lipid extractions were performed within 15 min of thawing and extracts stored at −80°C. Lipformed using a Thermo QExactive Plus Orbitrap mass spectrometer.

### 2.2 Data collection of lifestyle factors

The assessed lifestyle factors included dietary pattern, alcohol drinking, smoking, and physical activity. The 18‐item food frequency questionnaire was adapted from the National Cancer Institute Diet History Questionnaire. The food items, portion sizes, and nutrient database for this food frequency questionnaire were constructed by using the US Department of Agriculture 1994–1996 Continuing Survey of Food Intakes by Individuals. The frequency is coded from 1 to 6 (1 = Almost every day year round; 2 = Occasionally [4–6 times per week]; 3 = Occasionally [1–3 times per week]; 4 = Occasionally [<1 time per week]; 5 = Rarely; 6 = Almost never).^10^

Participants were asked whether they had ever smoked in their lifetime to define ever smokers and never smokers. Ever smokers were asked if they currently smoke or whether and when they had stopped smoking. The number of cigarettes per day was recorded for current or former smokers. Alcohol intake was measured by type of drink (wine vs. beer and other spirits) and quantity (number of glasses/bottles per week). Physical activity was measured by regularity, type of exercise, and incidental physical activity such as housework and gardening. Questionnaires were administered face to face by research assistants.

The questionnaire was mostly completed by close relatives or carers. Any responses given by VaD patients were counter‐checked with relatives or carers subsequently.

### 2.3 Dietary inflammatory index

The EDII was developed by Tabung et al.^11^ using eight pro‐inflammatory components (red meats, processed meats, organ meats, other fish, eggs, sugar‐sweetened beverages, tomatoes, and refined grains) and eight anti‐inflammatory components (leafy green vegetables, dark yellow vegetables, fruit juice, oily fish, coffee, tea, wine, and beer or other alcohol beverages). The scoring system of the EDII was designed using the Mediterranean diet pyramid^12^ and other literature.^13^ Each pro‐inflammatory component was scored 0, 1, or 2 points, and anti‐inflammatory components were scored 0, −1, or −2 points. To quantify how pro‐inflammatory or anti‐inflammatory each participant's diet was, we scored each component of our FFQ using the EDII (Table S1). Food groups from our FFQ that matched perfectly with those in the EDII include red meat, fatty meats, egg, offal, tea, coffee, wine, and other alcoholic beverages. Food groups from our FFQ that were not included in the EDII include beans, curry, cooked foods, deep‐fried foods, and full‐fat dairy. Food groups from the EDII that were not screened by our FFQ include sugar‐sweetened beverages and tomatoes. Some food groups from our FFQ partially matched with those in the EDII: The “fresh vegetables” category from our FFQ included both “leafy green vegetables” and “dark yellow vegetables” from the EDII; the “fresh fruit” category from our FFQ was scored as “fruit juice” from the EDII; the “fish” category from our FFQ included both “oily fish” and “other fish” from the EDII; the “bakery” category from our FFQ was scored as “bread” from the EDII. Where our FFQ's frequency categories did not match exactly with those in the EDII, probability weighted scores were given based on the EDII. Table S1 shows the final scoring system we used. Total scores ranged from −8 to +8, with a higher score indicating a higher inflammatory potential.

### 2.4 Statistical analysis

All collected data were recorded into a Microsoft Access 2007 database. The data were extracted and analyzed using STATA 16 (StataCorp). Continuous variables were presented as mean values with standard deviation. Dichotomous variables were presented as numbers and percentages. Nonparametric Mann‐Whitney *U* test and Fisher's exact test were used to compare demographic differences between the groups. Ordered logistic regression was used to compare differences in dietary habits between subjects with VaD and healthy controls, and covariate adjustment was performed for age, education, and socioeconomic status. Nonparametric series regression was used to compare EDII between subjects with VaD and healthy controls, and covariate adjustment was performed for age, education, and socioeconomic status. A *P* value of <0.05 was considered statistically significant.

To determine any association between plasma lipid profiles and dietary intake, we made nine nonparametric series regression models, one for each lipid group. The dependent variable was the lipid group, and the primary predictor variables were the consumption frequency of various food groups such as fresh fruit and vegetables, fish, diary, and deep‐fried foods. Covariate adjustment was performed for smoking status, alcohol drinking status, diabetes, age, education, socioeconomic status, and whether the participant had VaD or not. The dietary variables were collapsed into two frequency groups: regular (daily to weekly) versus infrequent (less than weekly/rarely/almost never). A stricter *P* value of <0.003 for statistical significance was used in view of the large number of covariates in these nine models.

## 3 RESULTS

### 3.1 Dietary and lifestyle factors

A total of 150 individuals were interviewed. General characteristics of the study population are presented in Table 1. The mean age of VaD participants (*n* = 36, 81.8 years, ranging from 68 to 94 years) was 2.5 years younger than the control group (*n* = 11, 84.3 years, ranging from 72 to 106 years) with *P* = 0.019, 95% CI: −4.551 to −0.431. There were 63.9% of VaD participants who had a lipid disorder compared to 43% of control participants (*P* = 0.036). Participants in the control group had greater educational attainment than participants in the VaD group (*P* < 0.001). Otherwise, the control and VaD groups were well matched in terms of other risk factors (body mass index, socioeconomic status, smoking and drinking status, presence of diabetes, and hypertension). Participants recruited from the War Memorial Hospital were all controls. VaD participants reported eating more deep‐fried foods [odds ratio (OR) = 16.747, 95% CI: 2.153–130.240, *P* = 0.007] and full fat dairy (OR = 2.7, 95% CI: 1.021–7.139, *P* = 0.045) compared to control (Table 2). VaD participants were less likely to perform regular exercise (OR =0.080, 95% CI: 0.023–0.283, *P* < 0.001), meet with friends and families (OR = 0.012, 95% CI: 0.001–0.127, *P* < 0.001), and read novels/books (OR = 0.095, 95% CI: 0.029–0.306, *P* < 0.001) (Table 3). Listening to music, religious activities, and watching television or listening to the radio did not differ in prevalence between the control and VaD groups. Only a minority in both groups were current smokers (2.6% of controls and 2.8% of VaD subjects), but roughly half of each group had current alcohol use (52.6% of controls and 44.4% of VaD). There was no difference of statistical significance when smoking and alcohol drinking were assessed between VaD participants and control (Table 4).

**TABLE 1:** Demographic and selected clinical characteristics.

	Control (*n* = 114)	VaD (*n* = 36)	*P*
Age	84.3 ± 4.67	81.8 ± 5.57	0.019*
Gender			
Male	44 (38.6)	16 (44.4)	0.562
Female	70 (61.4)	20 (55.6)	
Educational attainment			
Primary school	16 (14.0)	17 (47.2)	0.001**
Secondary school (year 7–11)	77 (67.5)	16 (44.4)	
Completed secondary school (HSC)	2 (1.8)	2 (5.6)	
Completed tertiary school	19 (16.7)	0 (0)	
Socioeconomic status			
Domestic	12 (10.5)	2 (5.5)	0.59
Intermediate production and transport	2 (1.8)	0 (0)	
Laborer	10 (8.8)	8 (22.2)	
Manager and administrator	6 (5.3)	4 (11.1)	
Professional	18 (15.8)	2 (5.5)	
Tradesperson	23 (20.2)	1 (2.8)	
Clerical	43 (37.7)	6 (16.7)	
BMI	25.25 ± 4.18	25.5 ± 5.14	0.85
MMSE	29.4 ± 0.76	19.6 ± 4.70	0.001**
Lipid disorder	49 (43.0)	23 (63.9)	0.036*
Diabetes	18 (15.8)	11 (30.6)	0.057
Hypertension	65 (57.0)	26 (72.2)	0.120
Smoking status			
Current smoker	3 (2.6)	1 (2.8)	1.000
Past smoker	38 (33.3)	15 (41.7)	0.425
Alcohol use			
Current drinker	60 (52.6)	16 (44.4)	0.447
Past drinker	15 (13.2)	5 (13.9)	1.000

*Note*: Age, BMI, and MMSE data were shown as mean ± SD; other data were shown as numbers with percentages in the parentheses. *P < 0.05, **P < 0.01.

**TABLE 2:** Diet pattern and frequency, adjusted for age, education, SES.

Diet	*p*
Fresh fruit	0.291
Fresh vegetables	0.275
Red meat	0.371
Fish	0.870
Beans	0.798
Curry	0.202
Cooking oil	0.938
Fatty meats	0.057
Deep fried	0.007**
Bakery	0.324
Full fat dairy	0.045*
Egg	0.069
Offal	0.995
Tea	0.769
Coffee	0.240

*Note*: *P < 0.05, **P < 0.01.

**TABLE 3:** Lifestyle activities, after adjusting for age, SES, education.

Life activities	OR	95% CI	*P*
Regular exercise	0.080	0.023 to 0.283	<0.001**
Meeting friends	0.012	0.001 to 0.127	<0.001**
Reading	0.095	0.029 to 0.306	<0.001**
Music	0.542	0.187 to 1.567	0.258
Religious	0.860	0.303 to 2.440	0.777
TV/radio	1.194	0.126 to 11.295	0.877

*Note*: **P < 0.01.

**TABLE 4:** Smoking and drinking habits.

Life activities	Group		OR	95% CI	*P*
	Control	VAD			
Current smoker	3 (2.6)	1 (2.8)	0.946	0.095 to 9.387	1.000
Past smoker	38 (33.3)	15 (41.7)	0.700	0.325 to 1.510	0.425
Current drinker	60 (52.6)	16 (44.4)	1.389	0.654 to 2.950	0.447
Past drinker	15 (13.2)	5 (13.9)	0.939	0.316 to 2.793	1.000

### 3.2 Association between diet and lipid profiles

Individuals who regularly consumed fatty meats had higher levels of phosphatidylcholine (205.897 units, *P* < 0.001, 95% CI: 102.172 units to 309.622 units), phosphatidylethanolamine (19.772 units, *P* < 0.001, 95% CI: 12.499 units to 27.044 units), and phosphatidylinositol (73.343 units, *P* < 0.001, 95% CI: 61.328 units to 85.358 units) compared to those who ate fatty meats infrequently (Figure S1). Individuals who regularly consumed deep‐fried food had lower levels of phosphatidylcholine (−228.429 units, *P* < 0.001, 95% CI: −315.120 units to −141.738 units) and phosphatidylinositol (−68.083 units, *P* < 0.001, 95% CI: −80.432 units to −55.734 units) compared to those who ate deep fried food infrequently. Individuals who regularly consumed bakery products had higher levels of phosphatidylinositol (6.526 units, *P* < 0.001, 95% CI: 2.747 units to 11.158 units) compared to those who ate bakery products infrequently. We did not find any association between age and sex with plasma lipid profiles. However, male participants are more likely to eat beans daily compared to females (OR =3.59, 95% CI: 1.08 to 11.97, *P* = 0.037).

### 3.3 Pro‐inflammatory diet and risk of vascular dementia

**FIGURE 1:**
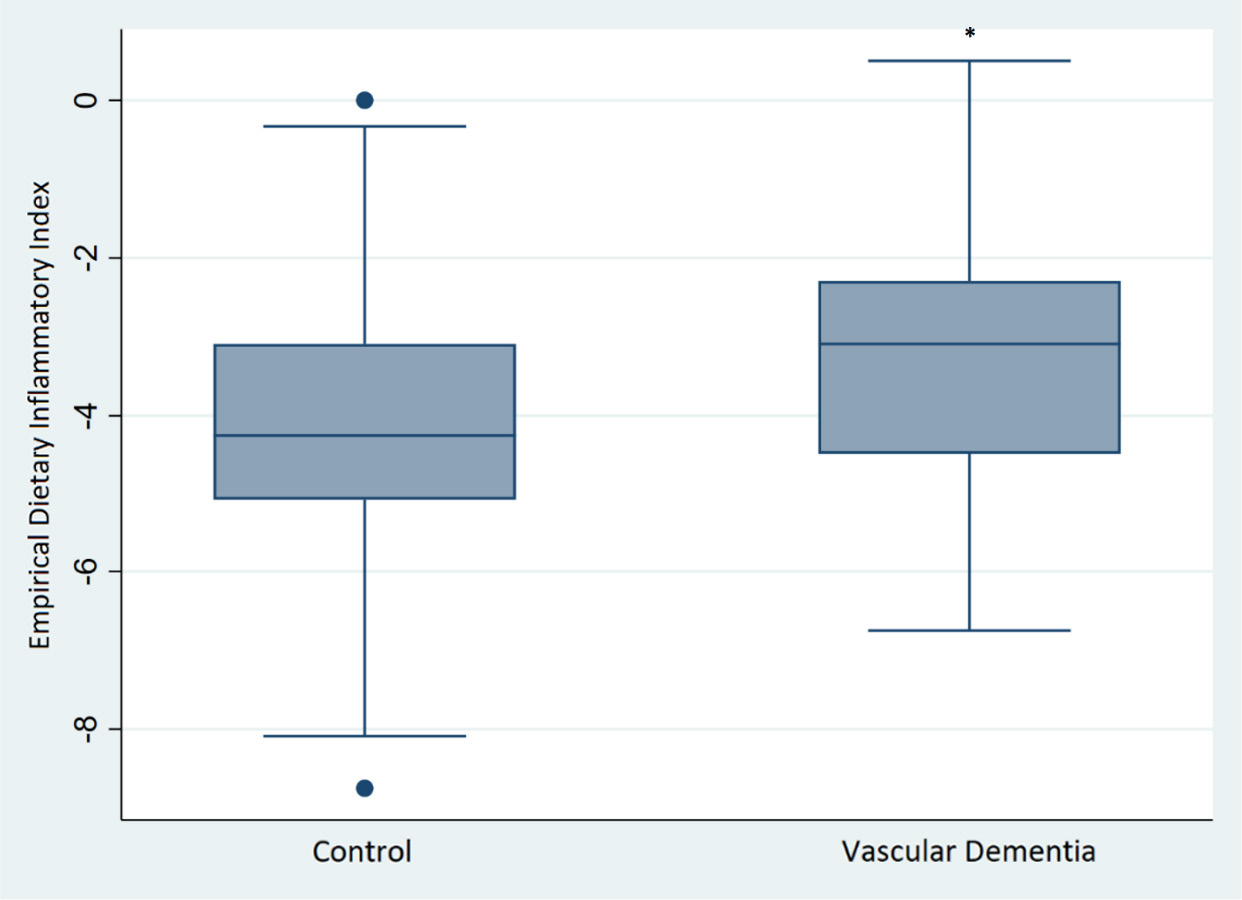
Comparison of the Empirical Dietary Inflammatory Index (EDII) of control group and vascular dementia group.

The mean EDII score in the control group was −4.03, and the mean EDII score in the VaD group was −3.24 (Figure 1). After adjusting for age, education, and SES, there was no statistically significant difference in mean EDII between the control and VaD group.

## 4 DISCUSSION

### 4.1 Lipid disorders

We found dyslipidemia was more prevalent among VaD participants. Elevated low‐density lipoproteins have been previously reported to increase the risk for VaD in stroke patients^14^, ^15^ and also affects amyloid β processing and deposition causing dementia, in particular Alzheimer's disease.^16^

### 4.2 Exercise

We found VaD participants do less exercise. Higher levels of physical activity seem to be protective for development of VaD, even in the presence of white matter changes, independent of age, education, and other risk factors.^17^ A systematic review found physical activity was associated with reduced risk of vascular dementia (OR = 0.62, 95% CI: 0.42–0.92) when analyzing five studies with 10,482 patients.^18^ A recent trial found that a multidomain intervention (including regular exercise) could improve or maintain cognitive functional abilities in the elderly.^19^, ^20^ Our observational study appears to support the idea that exercise is protective against VaD, as regular exercise is less prevalent in the VaD group (OR 0.080). However, our result needs to be interpreted with care since people with VaD might have less capacity to exercise.

### 4.3 Education

We found VaD participants engage less frequently in social interaction and educational or reading activity. Lower educational level is associated with increased prevalence of VaD.^21^, ^22^ Participation in late life cognitive activities may be a better marker of cognitive reserve than the educational level attained earlier in life.^21^ Higher educational level may be protective for motoric function, even in the presence of white matter hyperintensities. Participants with higher education had better motor function, but still had a degree of decline in motoric symptoms.^23^ These results are consistent with the passive cognitive reserve hypothesis.^24^, ^25^ A study found that there was a delay in onset of dementia in bilingual patients, even in those who are illiterate, in different dementia subtypes.^26^ Another study found that factors of life experience and learning other than years of schooling may be protective for the development of cognitive impairment.^27^ Recognizing the role of prior and continuing educational activities in cognitive impairment has an importance for the prevention or maintenance of cognitive function as well as the determination of the clinical diagnosis of VaD or other dementia.^27^ Our observational study appears to suggest cognitive activities such as socialization with families and friends and reading are protective against VaD, as regular participation in these activities are less prevalent in VaD subjects (OR 0.012 and OR 0.095, respectively). However, people with VaD may have less motivation and cognitive ability to perform these tasks, so our results need to be interpreted with care.

### 4.4 Diet

In our study, VaD participants reported more frequent consumption of deep‐fried food and full‐fat dairy (Table 2) despite adjusting for education and socioeconomic status. Observational studies have identified certain dietary components that may affect the risk of vascular dementia, but randomized controlled trials would be needed to prove their efficacy.^28^ Higher dietary intake of saturated fats, trans‐unsaturated fats, or cholesterol may increase the risk of dementia.^29^

Higher fish and DHA consumption are associated with lower risk of vascular dementia, cognitive decline, and less development and progression of white matter hyperintensities. Omega 3 polyunsaturated fatty acid (PUFA) therapy is hypothesized to promote brain health by supporting the small blood vessels in the brain.^30^, ^31^ Flavonoids, particularly berries, possibly have more antioxidants and may be protective for cognitive impairment.^32^, ^33^ Regular cocoa consumption has been shown to improve neurovascular coupling and cognitive function and better neurovascular coupling is associated with greater white matter structural integrity.^34^ Dietary soy isoflavone supplementation does not appear to be protective.^35^

Ceramides have been reported to regulate the effect of insulin on skeletal muscle and increased levels of ceramide have been reported in obese subjects with type II diabetes.^36^ However, lower levels of ceramides have also been associated with increased demyelination^37^ and a higher risk of VaD.^38^ Bakery products such as whole grain bread and cereals are poor in refined carbohydrates and therefore may inhibit hepatic ceramide synthesis and export. However, offal products are rich in ceramide content and hence increased consumption of offal‐based products can increase plasma ceramide levels. The FRUVEDomic pilot study showed that a diet rich in fruits and vegetables and low in refined carbohydrates improved inflammatory status, which correlated with ceramide levels in in young adults.^39^

We recently found significantly higher levels of diglycerides, particularly DG (12:0/20:5) and DG (18:0/18:0) in the plasma of VaD subjects.^38^ Mono‐ and diglycerides, which are present in most breads and baked products, are formed by chemically joining glycerol to fatty acids from animal fats or vegetable oils. These lipids are used as emulsifiers, preventing breads and baked products from crumbling, or becoming stale, and/or maintaining oil and water components in the required viscosity (e.g., in salad dressings). It has been estimated that the percentage of vegetable oil–sourced mono‐ and diglycerides used in the United States is approximately 70%.^40^ Diglycerides also act as emulsifiers in alcoholic beverages such as beer and wine and are key ingredients in coffee whiteners. This may likely explain the observed increases in diglycerides in regular alcohol and coffee consumers.

### 4.5 Dietary inflammatory index

Other studies have reported associations between dietary intake, inflammation, cognitive performance, and risk of dementia, but ours is the first to study specifically VaD. For example, the Supplementation en Vitamines et Minerauz Antioxydants Study reported that higher DII scores at midlife were associated with cognitive decline by at least 13 years later.^41^ The Whitehall II study used a set of predefined foods to identify an association between inflammatory dietary intake and IL‐6. More specifically, the study found a correlation between higher intake of red meat, processed meat, peas, legumes, and fried food, and lower consumption of whole grains increased inflammatory potential and accelerated cognitive decline.^42^ Other diets, e.g. the Mediterranean and DASH diets have demonstrated lower inflammatory potential. These diets are rich in polyphenols, antioxidants, and anti‐inflammatory agents that can lower the inflammatory potential.^43^, ^44^ However, not all studies have promising results. For example, the WHIMS found no significant association between healthy dietary patterns and cognitive decline.^45^ Likewise, our study did not find a significant difference in EDII between VaD subjects and healthy controls. Contradictory findings may be attributed to differences in study populations, methodologies, or dietary scoring methods.^46^, ^47^ Insufficient power could also contribute to a negative result. Sample size calculations using the expected difference in EDII from our study found that 68 VaD subjects and 68 healthy controls will be required to reach statistical significance.

### 4.6 Strengths and weaknesses

The strength of our study is that we looked into protective factors that are unexplored in vascular dementia and healthy aging.^48^ We also ascertain our cases and controls. We were able to classify clinically, not just purely based on radiological appearances of leukoaraiosis, cases of VaD using current validated diagnostic methods.

Several limitations should be considered. First, information and data collection might be affected by recall bias. Participants with vascular dementia are more likely to be affected by recall bias than controls, since a high degree of memory and potentially literacy and numeracy skills are required to complete the study questionnaires. This limitation universally affects questionnaire‐based studies of people living with dementia. Secondly, we have no follow‐up data on changes in lifestyle factors over time, as this was a cross‐sectional questionnaire. With exception of past medical history, lifestyle, and clinical exposures have been obtained at study baseline. Given that the pathological changes of VaD can occur a decade or more before diagnosis it is likely that reverse causation could explain the lifestyle differences observed between the cases and controls. Thirdly, there were more female control participants than males. This might cause bias in lifestyle factors and educational activities because of disadvantaged societal upbringing and expectations for females in the early part of this century. Fourth, we cannot completely rule out confounding by unmeasured lifestyle factors or psychosocial symptoms (e.g., carer stress or depressive symptoms) that may be associated with VaD. Fifth, while the National Cancer Institute's Diet History Questionnaire is well validated,^49^ our adapted version has not been validated. It is possible that our 18‐item screener may not be sensitive enough to derive dietary patterns or detect diet‐disease relationships. Lastly, our study population is an Australian sample, and our findings may not be generalizable to other populations with different dietary habits.

## 5 CONCLUSION

Our study demonstrates a graded inverse association between healthy lifestyle factors and VaD in both men and women. A healthy lifestyle may be useful in the primary prevention of VaD, although further prospective studies are needed to confirm this.

## AUTHOR CONTRIBUTIONS

Jun Dai: literature review, design, data analysis and writing; Daniel Kam Yin Chan: conceptualisation, literature review, design and writing; Richard O Chan: literature review, data collection/analysis and writing; Vasant Hirani: design and writing; Ying Hua Xu: design, data collection, data analysis and writing; Nady Braidy: design, plasma lipid profiles and writing.

## CONFLICT OF INTEREST STATEMENT

Authors were employed by their affiliated organizations (Bankstown‐Lidcombe Hospital and University of New South Wales).

## ETHICAL APPROVAL AND CONSENT TO PARTICIPATE

This study has been approved by the South Western Sydney Local Health District Human Research Ethics Committee and institutional human ethics committees at each investigational site (Ethics registration number: HREC/16/LPOOL/190). All responses to questionnaires and blood samples were collected in accordance with the ethical guidelines mandated by the South Western Sydney Local Health District Human Research Ethics Committee. All individuals were over 18 years of age and were approached using approved ethical guidelines. All participants provided written consent. The study was conducted according to the Declaration of Helsinki principles.

## Supporting information


Figure S1.

Table S1.

## Data Availability

The data that support the findings of this study are available from the corresponding author on special request.
