# Supplementary material for: The association between dietary patterns, plasma lipid profiles, and inflammatory potential in a vascular dementia cohort
Source: Aging Med (Milton). 2023 Apr 1;6(2):155–62. doi: 10.1002/agm2.12249 (PMC10242272; doi:10.1002/agm2.12249)

**Supplemental figure 1. Average effects of lifestyle variables on plasma (A) ceramide, (B) cholesterol esters, (C) diacylglycerol , (D) lyso-phosphatidylcholines, (E) phosphatidylcholines, (F) phosphatidylethanolamines, (G) phosphatidylinositol, (H) sphingomyelin, and (I) triacylglycerols. Error bars indicate 95% confidence intervals.** Regression models developed using STATA 16 (StataCorp)

**(A)**


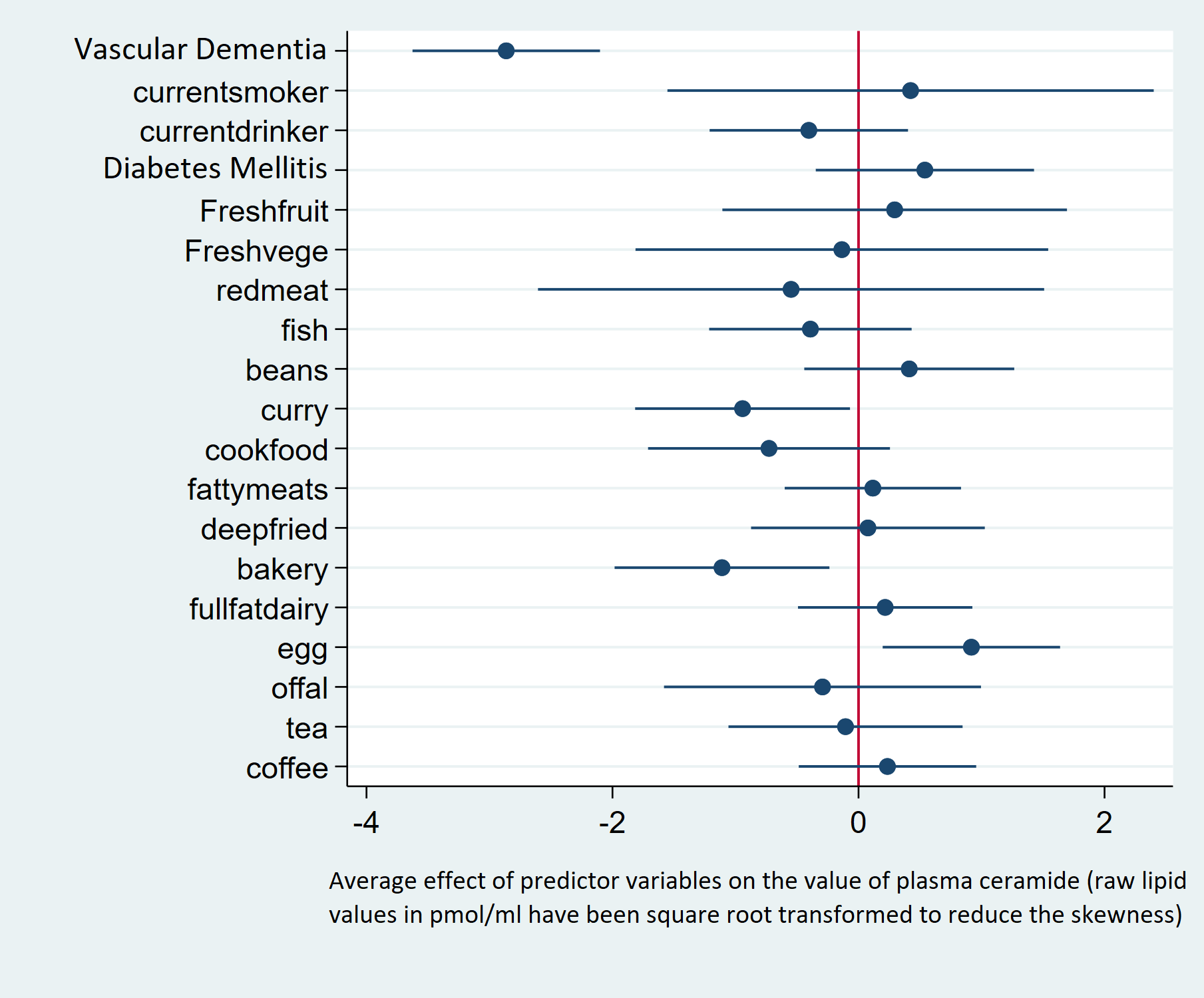


After adjusting for age, SES, education:


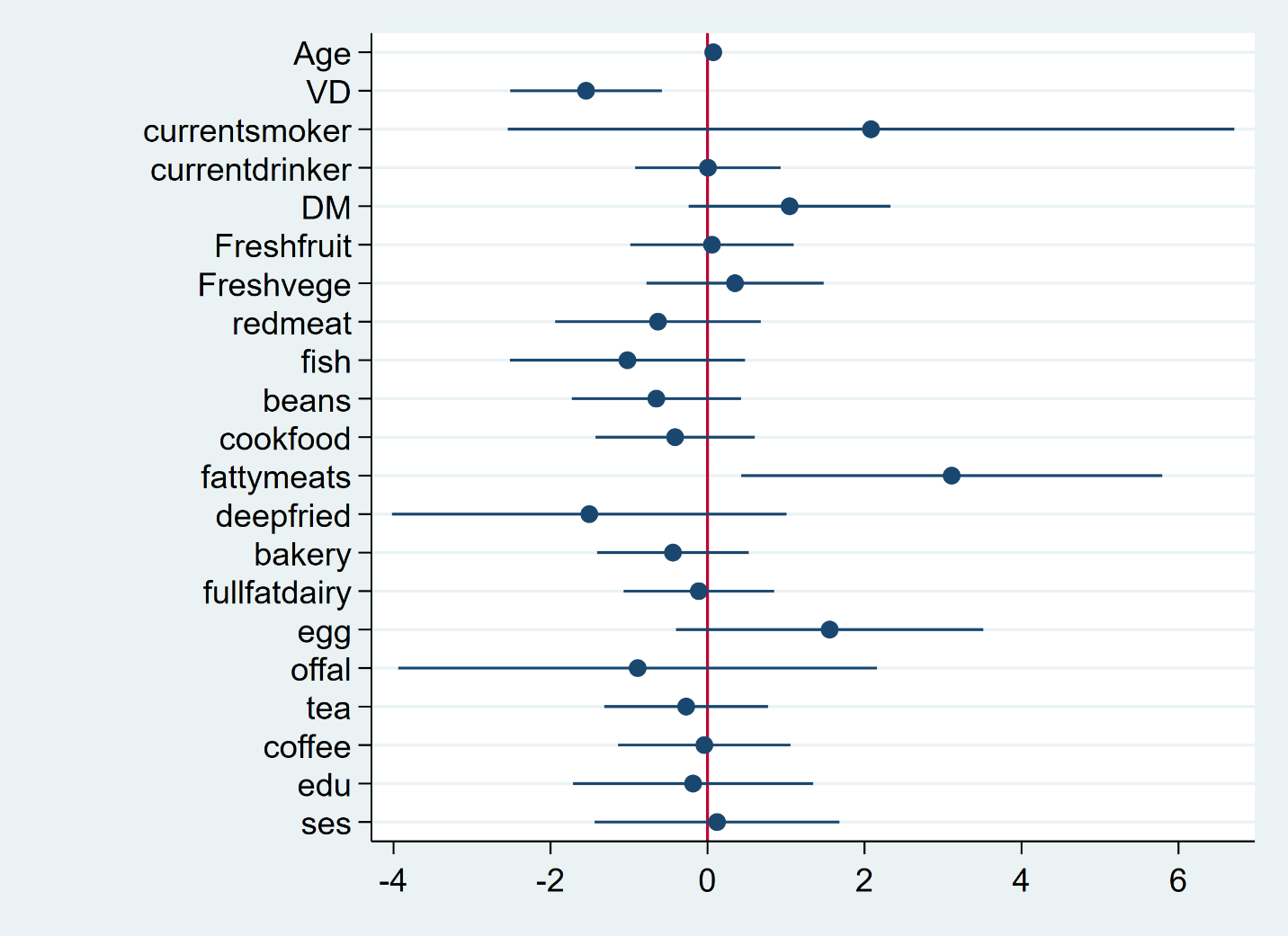


**(B)**


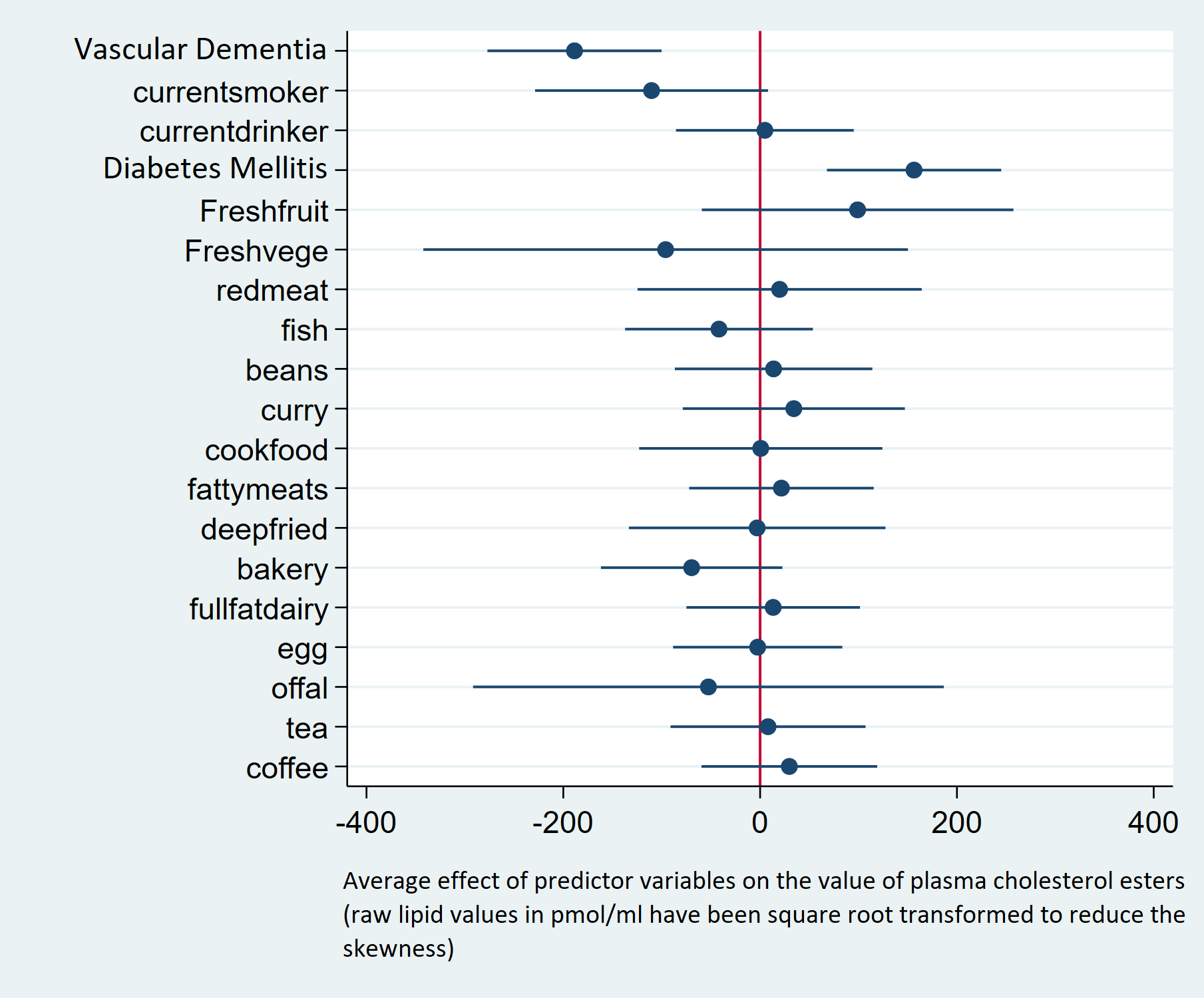


After adjusting for age, SES, education:


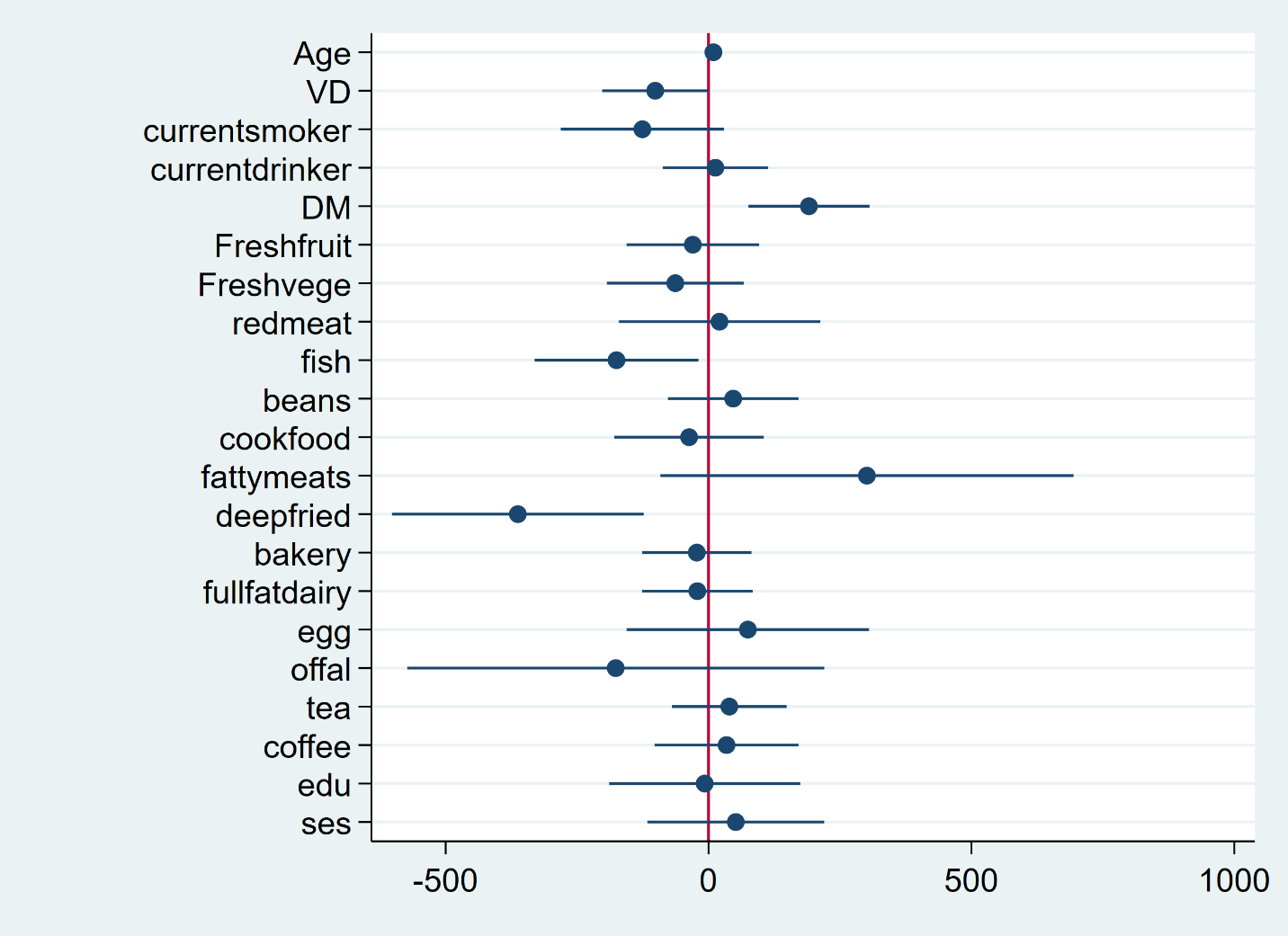


**(C)**


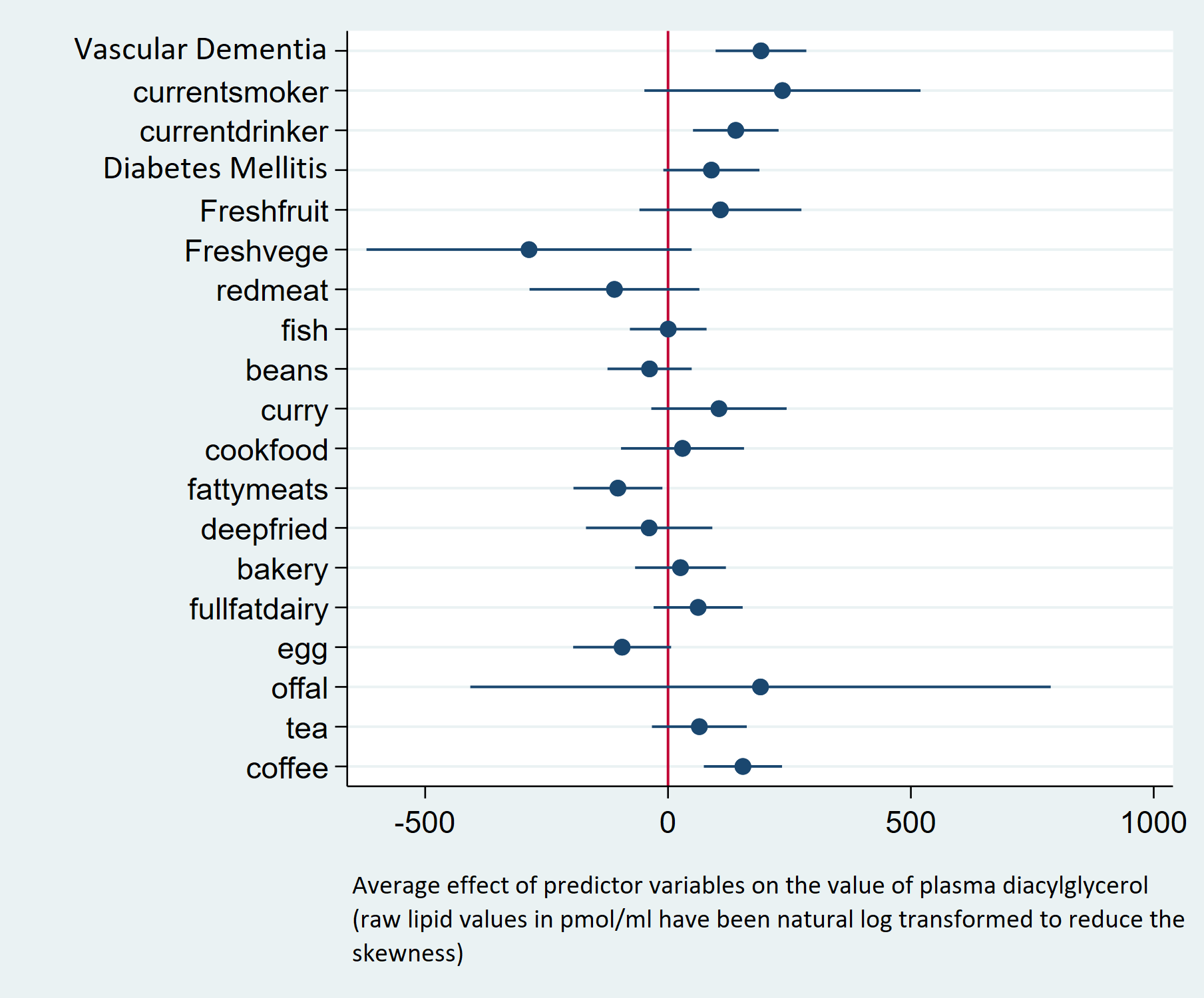


After adjusting for age, SES, education:


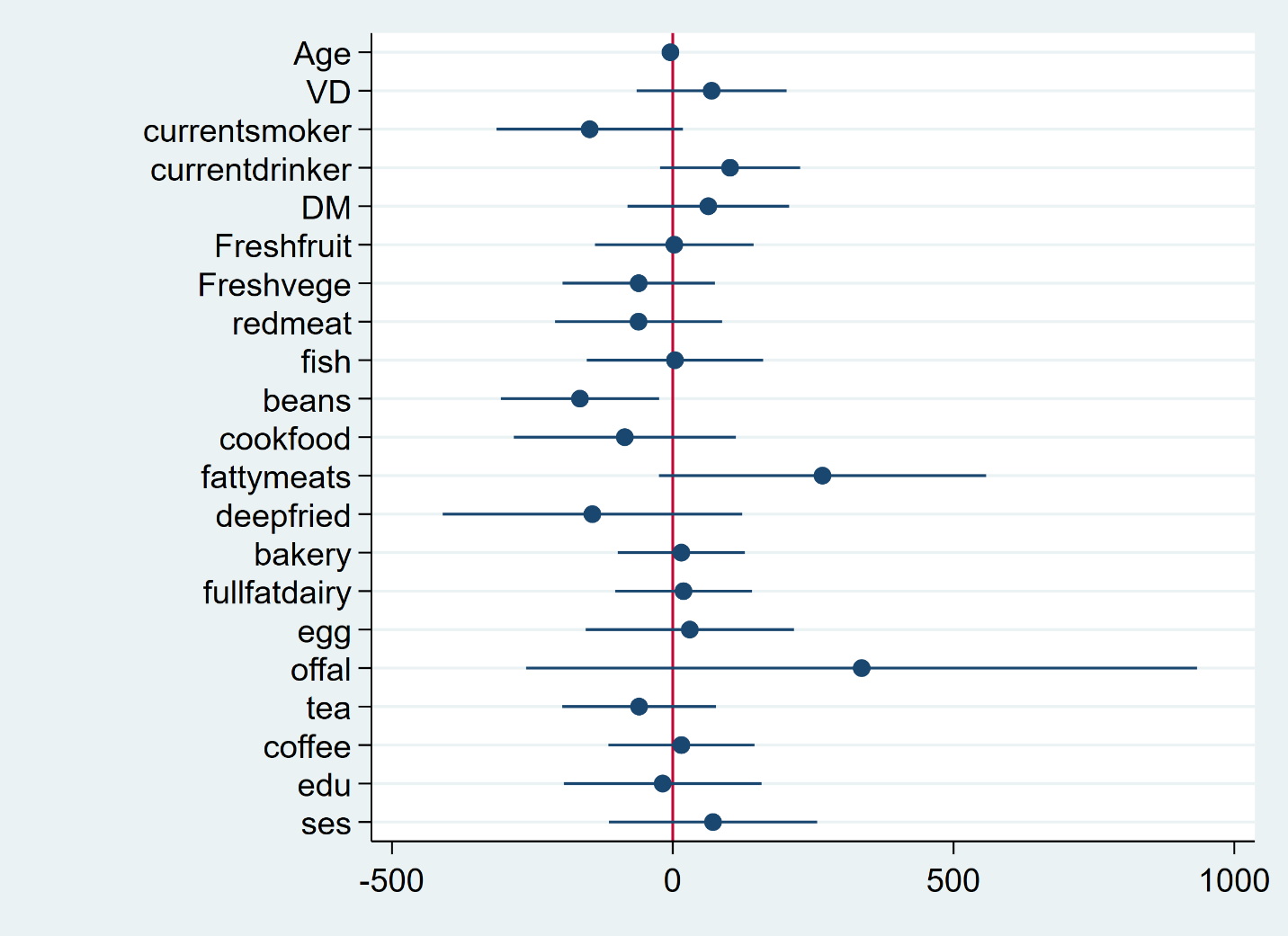


**(D)**


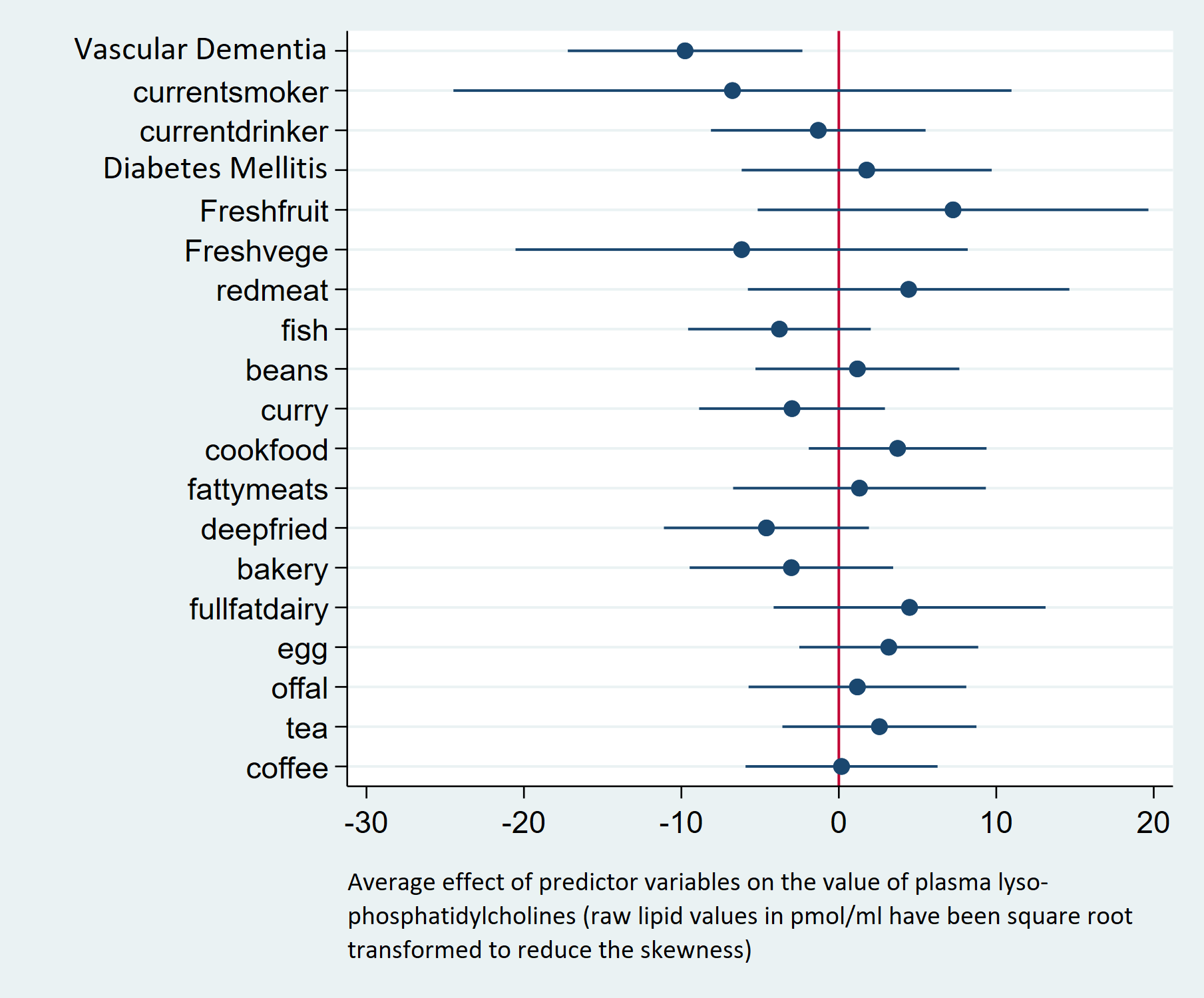


After adjusting for age, SES, education:


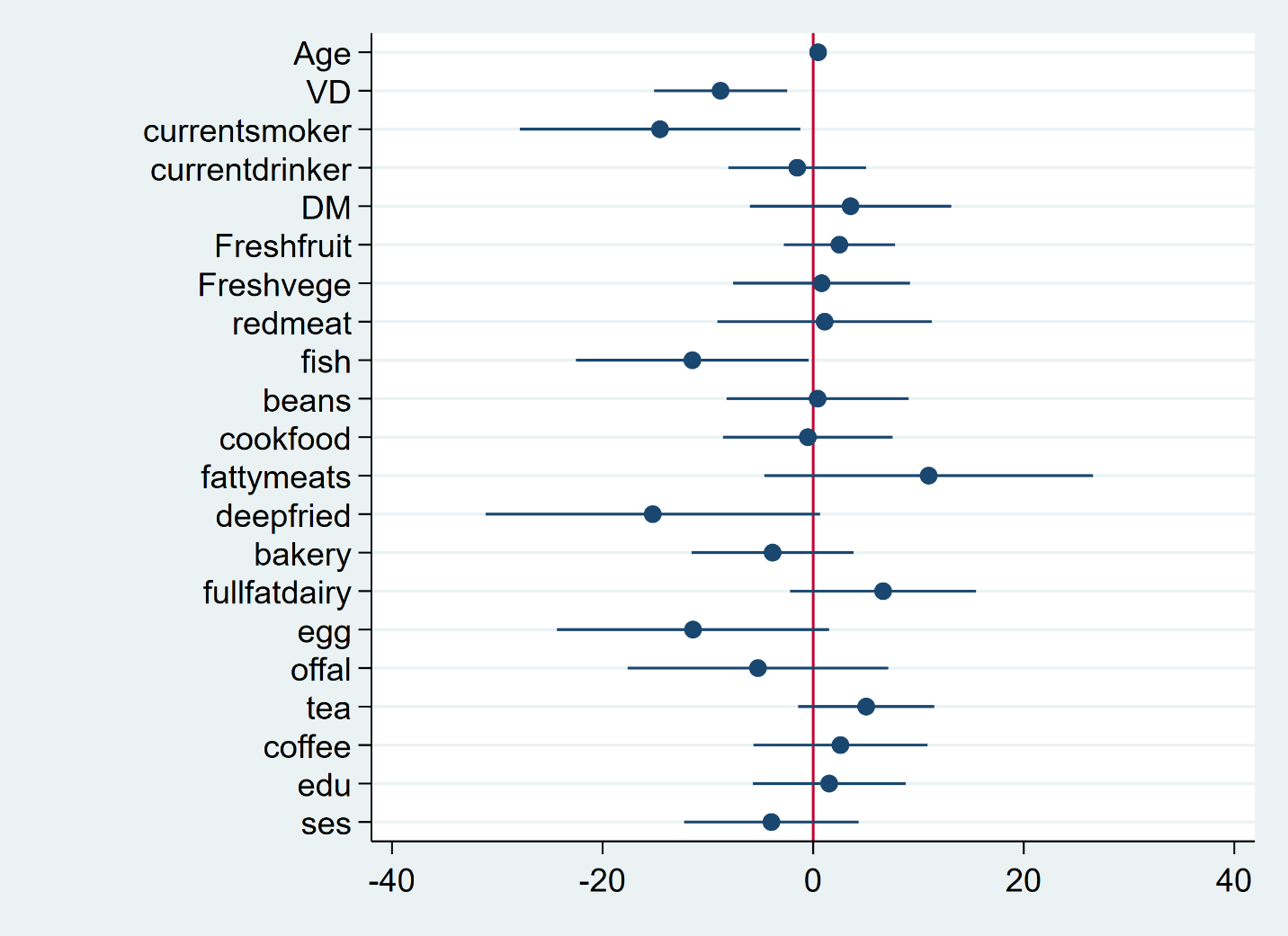


**(E)**


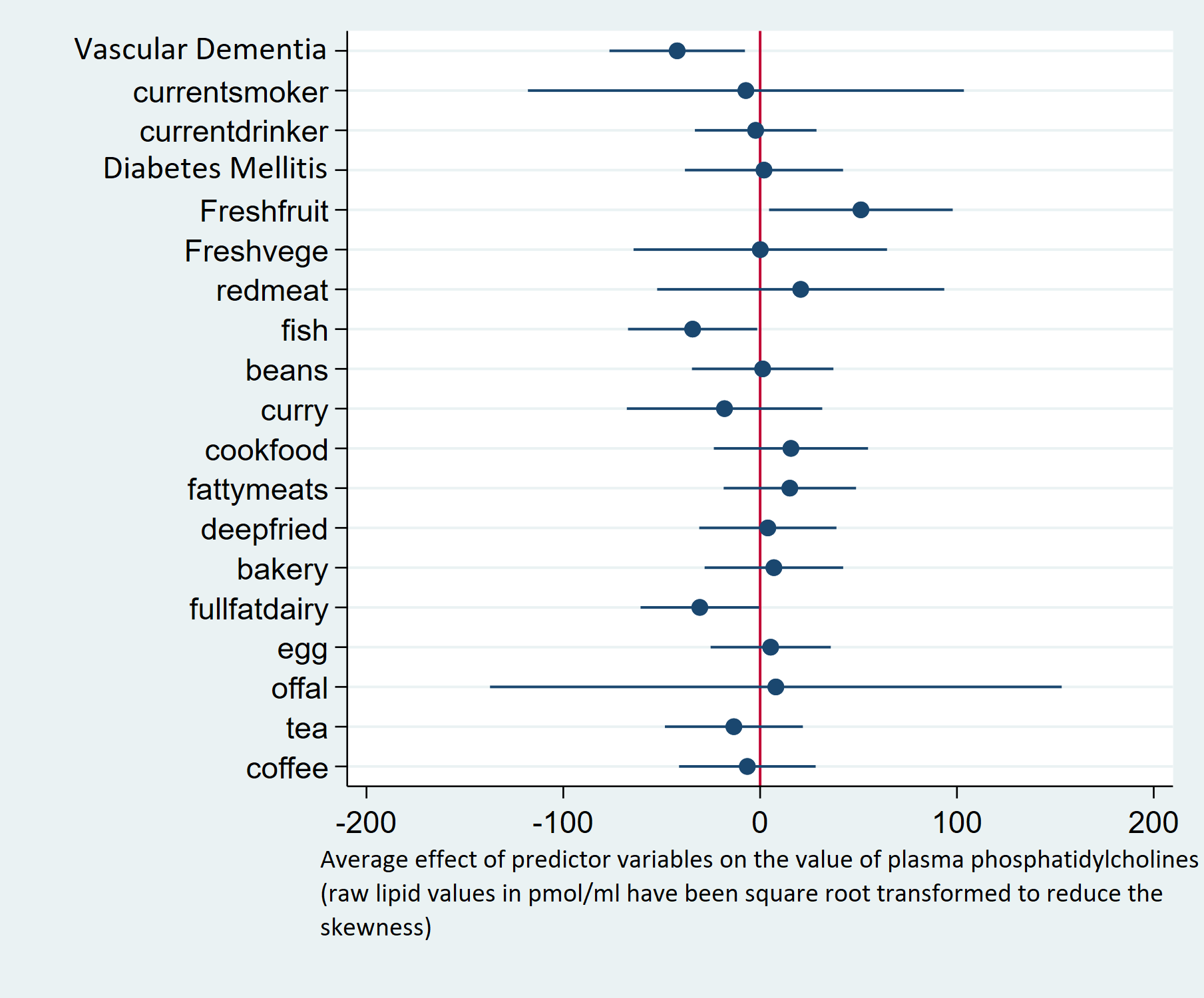


After adjusting for age, SES, education:


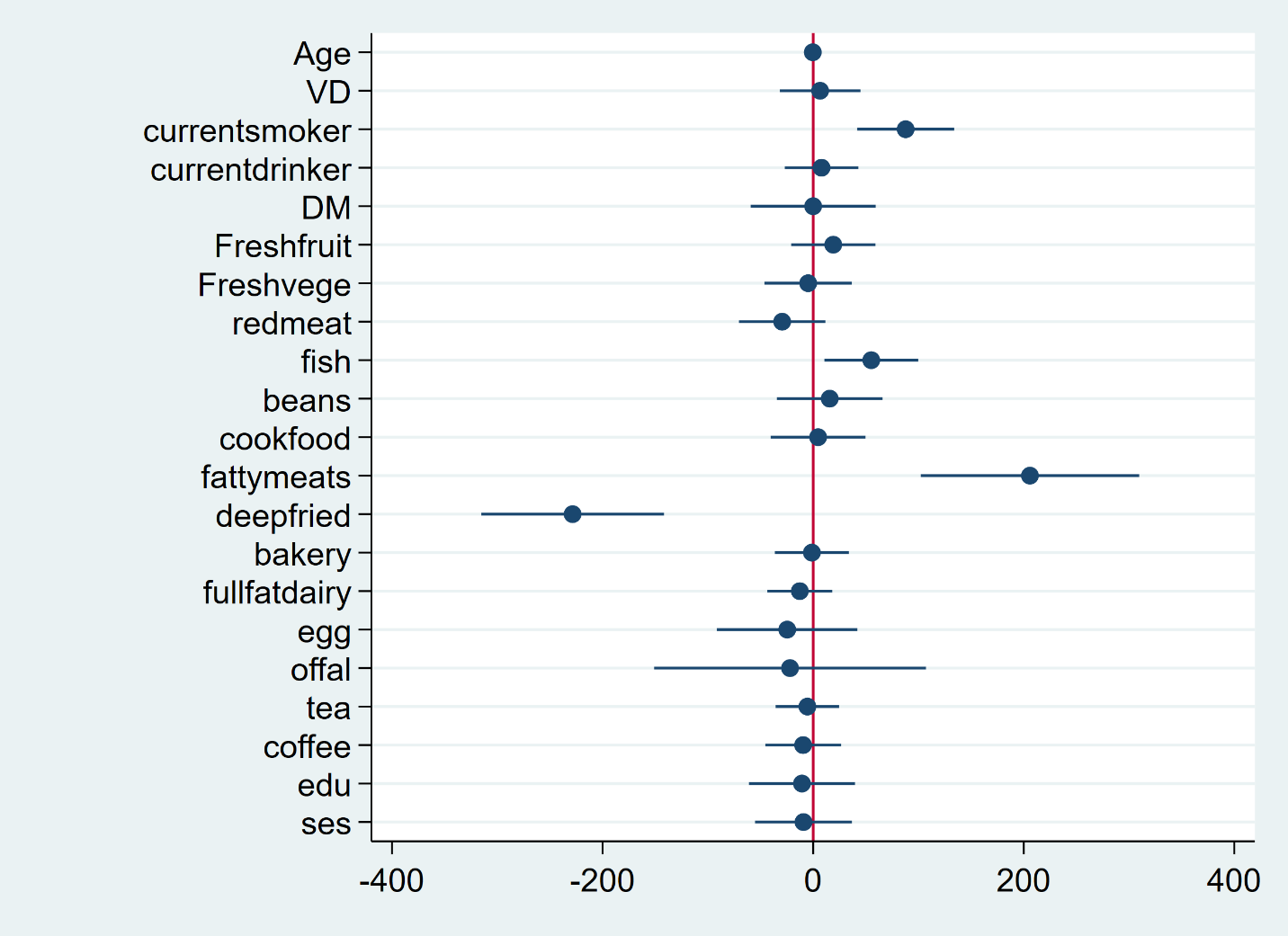


**(F)**


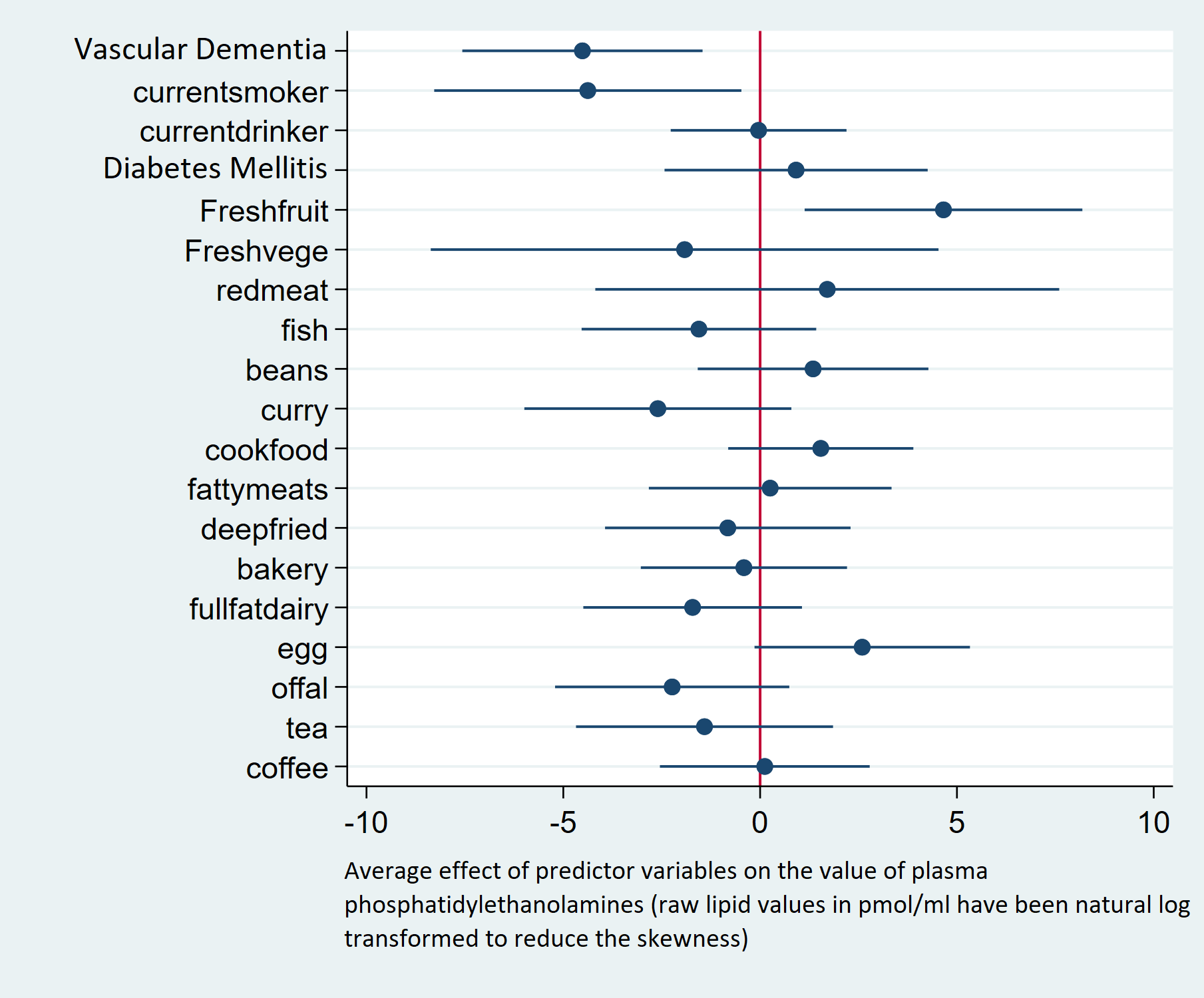


After adjusting for age, SES, education:


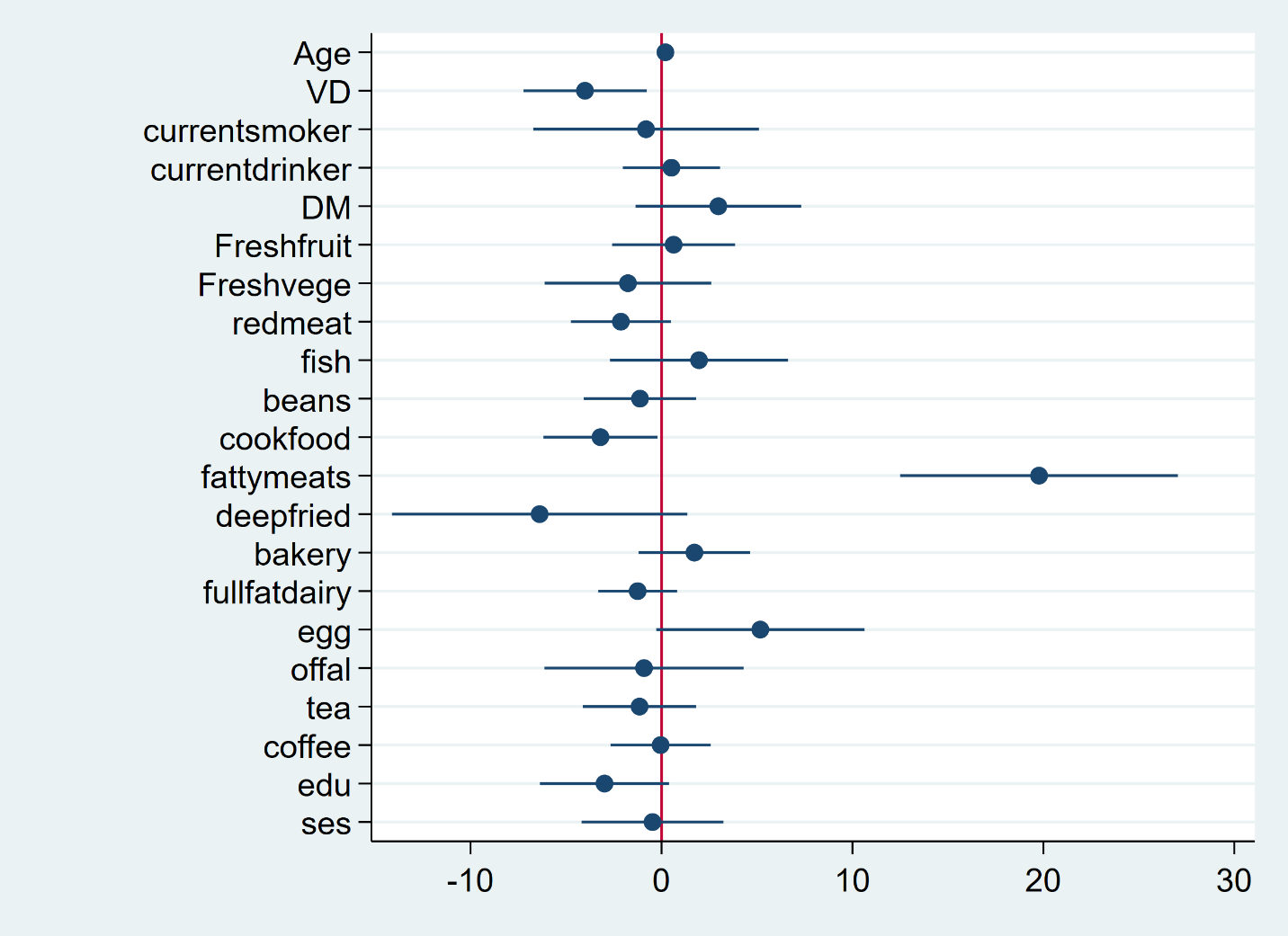


**(G)**


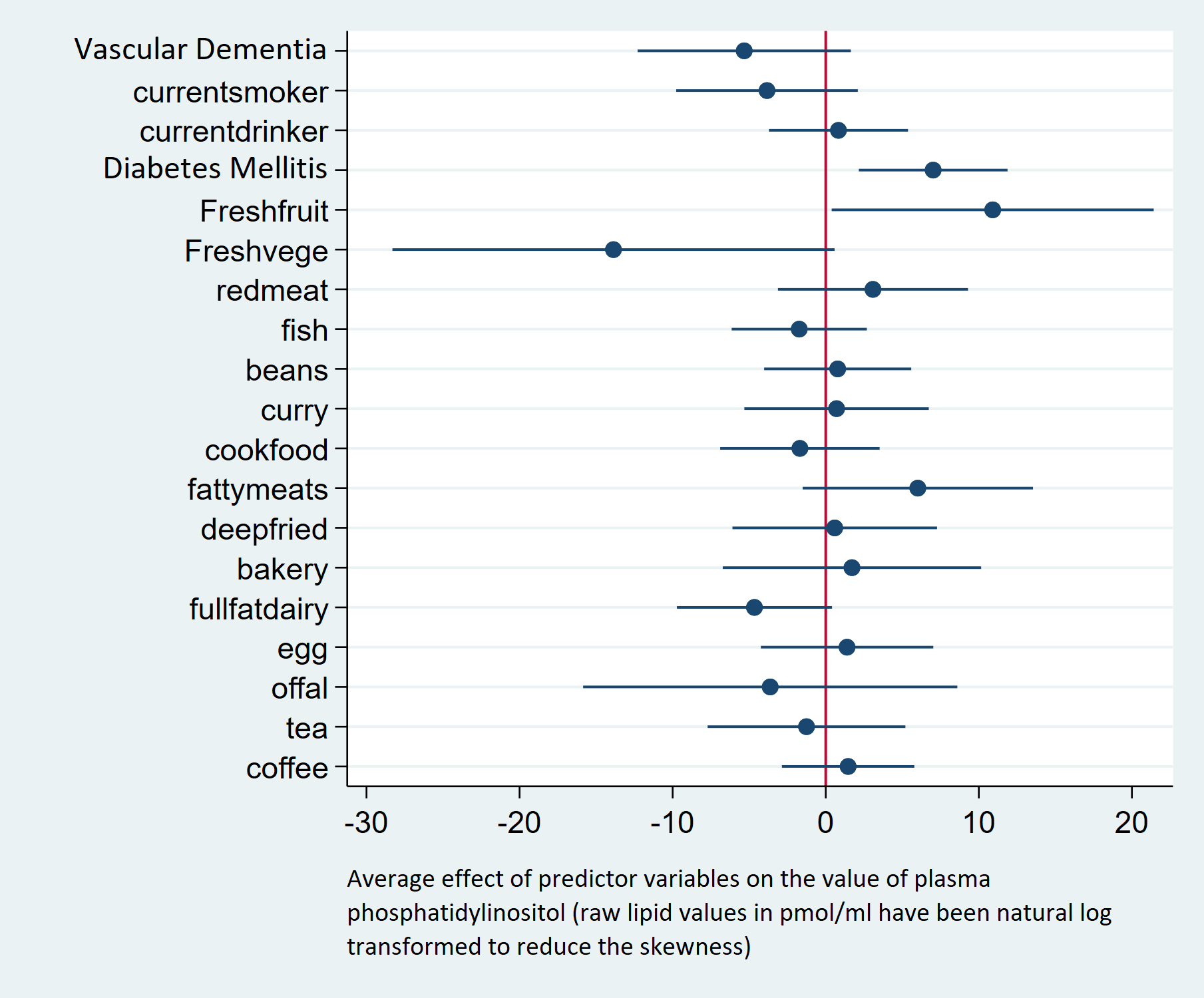


After adjusting for age, SES, education:


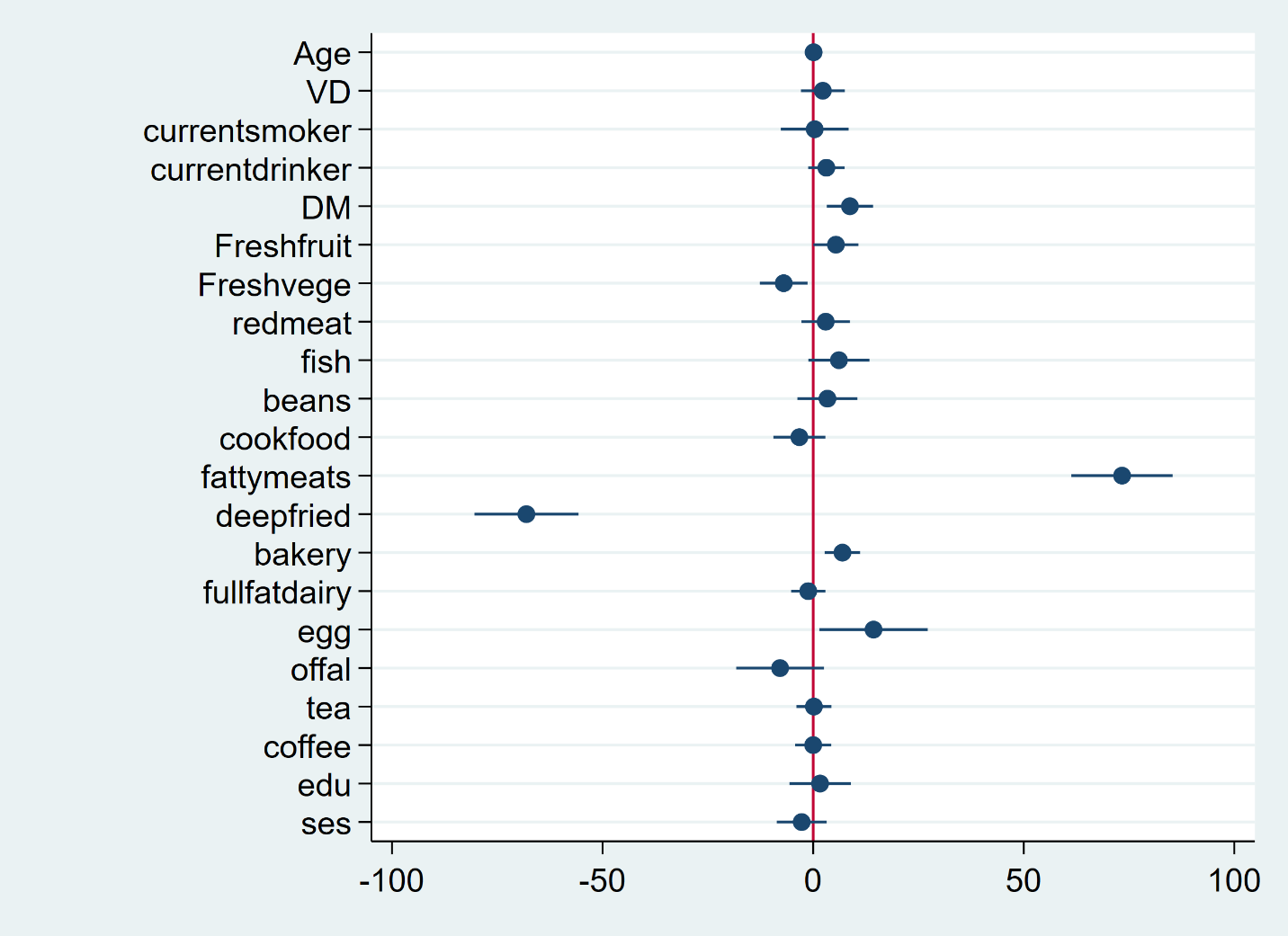


**(H)**


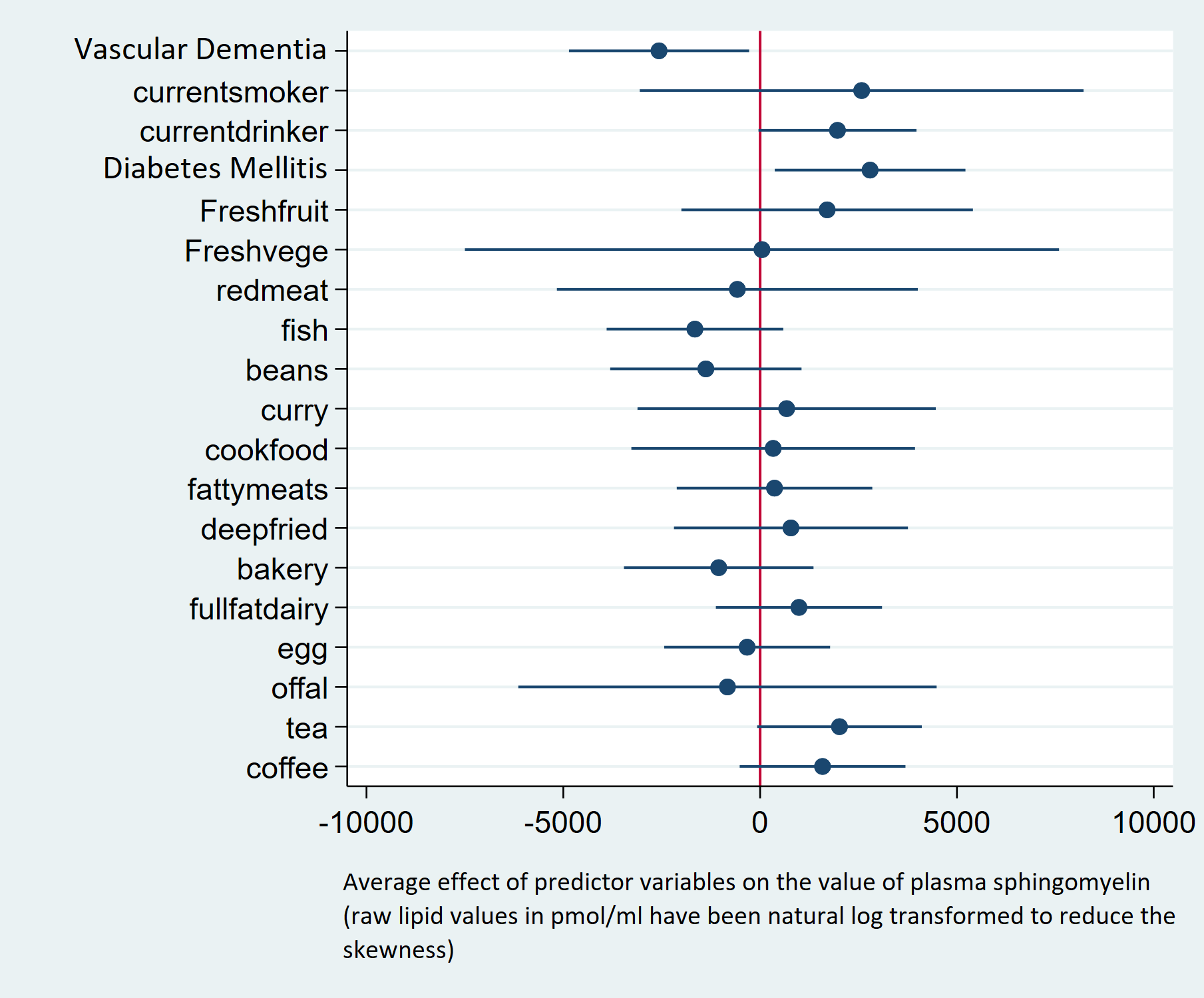


After adjusting for age, SES, education:


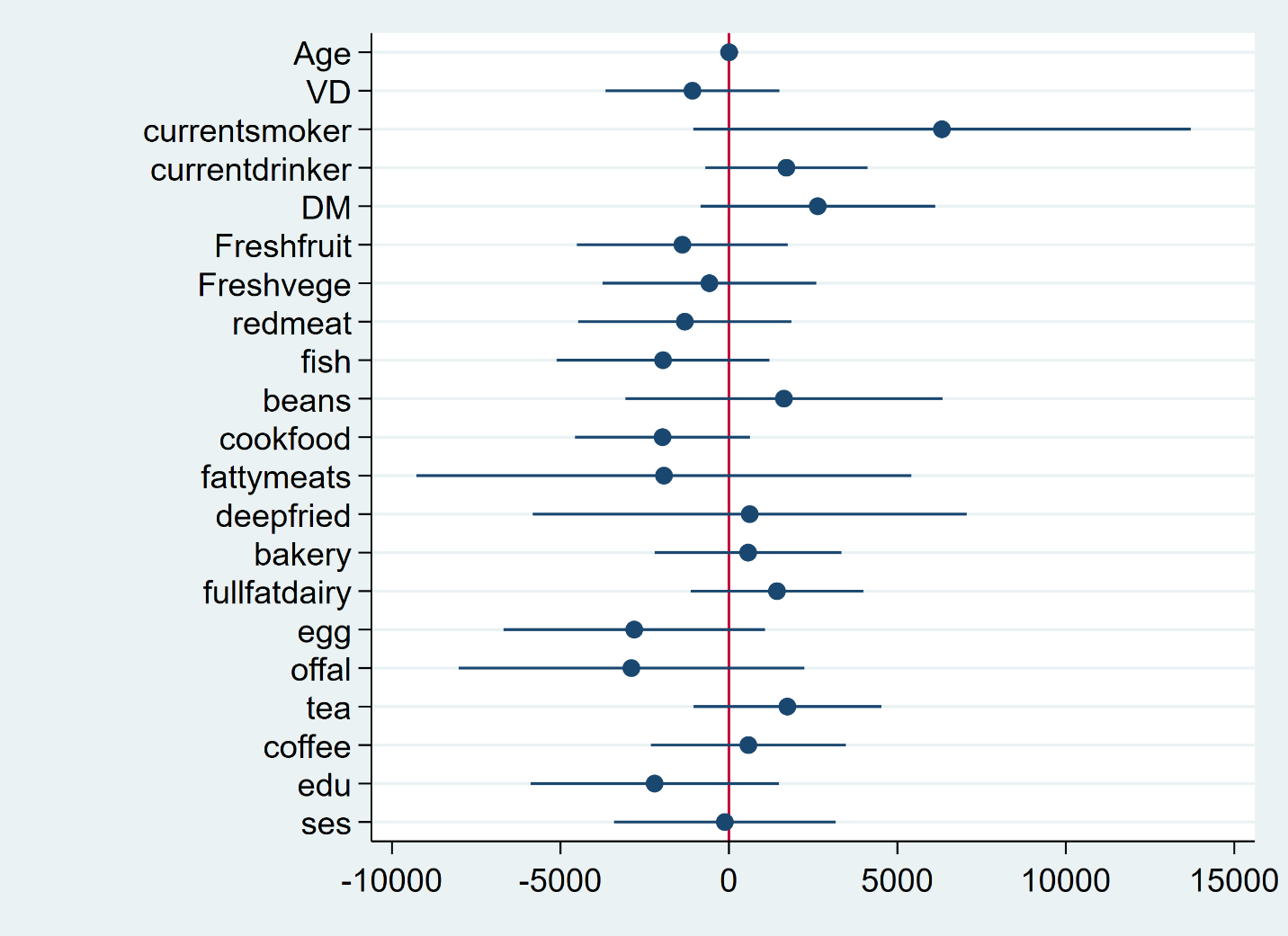


**(I)**


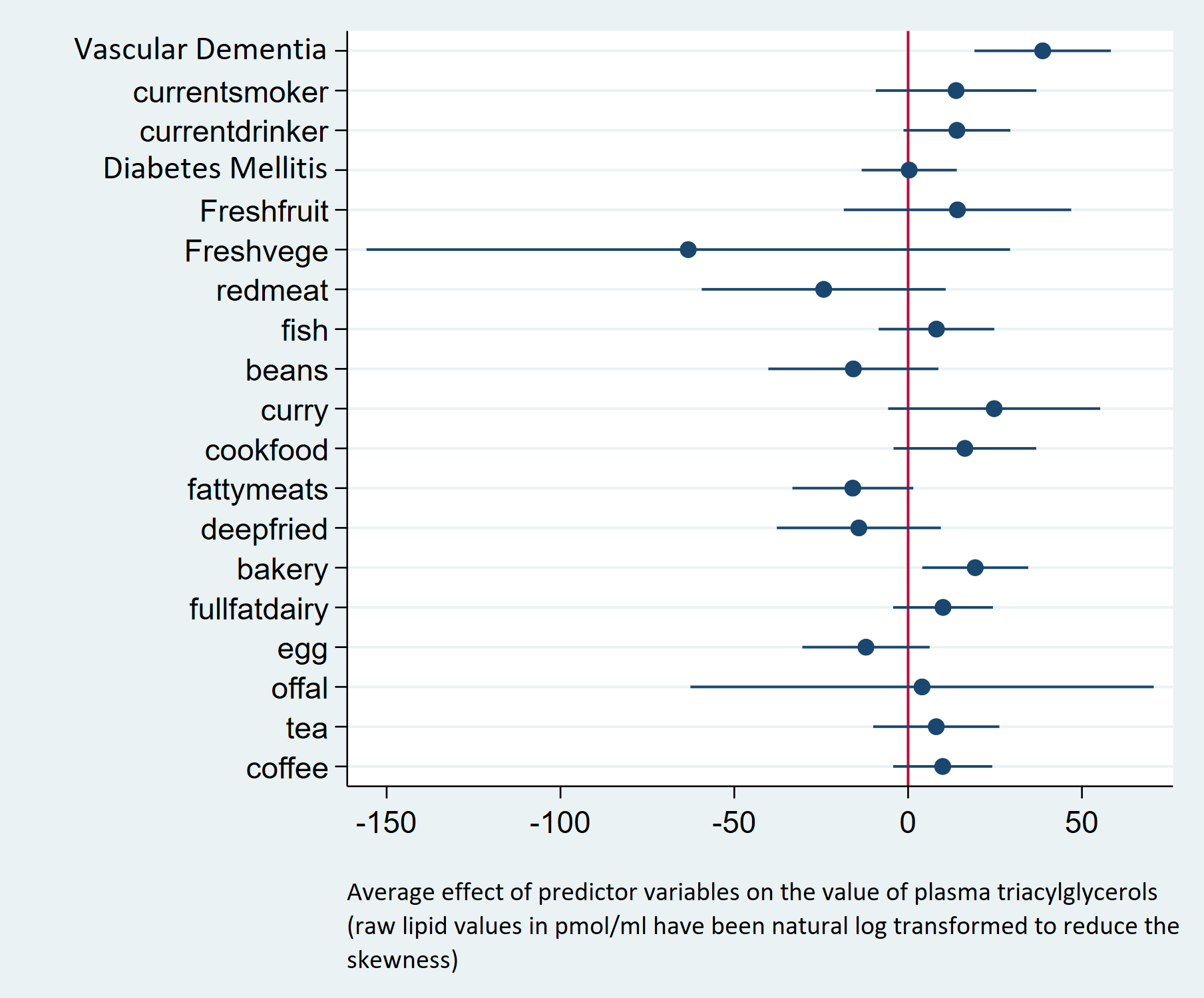


After adjusting for age, SES, education:


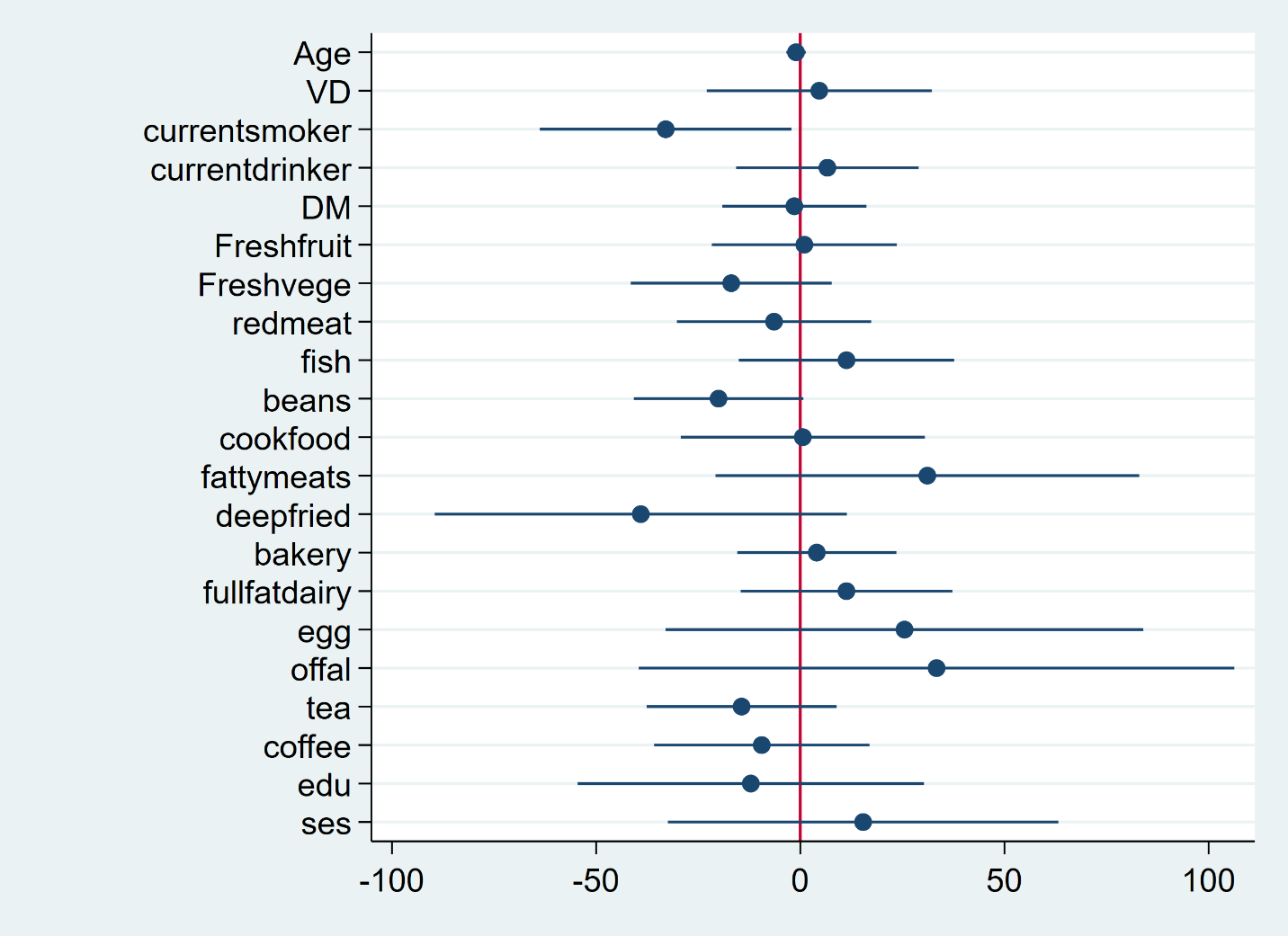

Supplement: Supplementary file 1 — Figure S1. [file AGM2-6-155-s001.docx]
