# Supplementary material for: The association between dietary patterns, plasma lipid profiles, and inflammatory potential in a vascular dementia cohort
Source: Aging Med (Milton). 2023 Apr 1;6(2):155–62. doi: 10.1002/agm2.12249 (PMC10242272; doi:10.1002/agm2.12249)

**Supplementary table 1: How the EDII scoring system has been applied to our Food Frequency Questionnaire.**


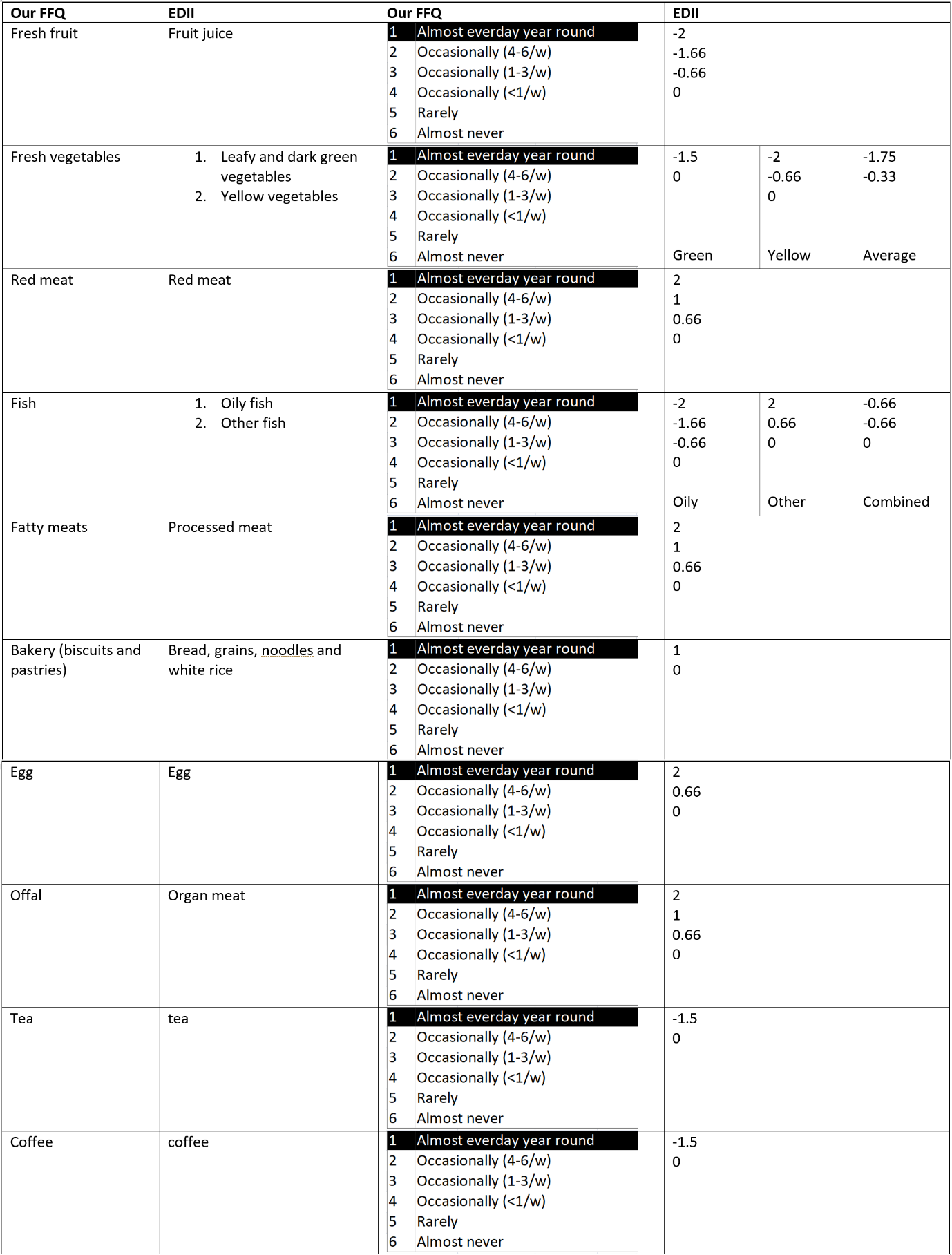

Supplement: Supplementary file 2 — Table S1. [file AGM2-6-155-s002.docx]
